# Orthopaedic Disorders in Cerebral Palsy in International Cooperation Projects: A Descriptive Cross-Sectional Study

**DOI:** 10.3390/ijerph18157872

**Published:** 2021-07-25

**Authors:** Elisa Maria Garrido-Ardila, Berta Caro-Puertolas, Maria Jiménez-Palomares, Jesús Montanero-Fernández, Trinidad Rodríguez-Domínguez, Juan Rodríguez-Mansilla

**Affiliations:** 1 ADOLOR Research Group, Department of Medical-Surgical Therapy, Medicine and Health Sciences Faculty, Extremadura University, 06006 Badajoz, Spain; egarridoa@unex.es (E.M.G.-A.); jrodman@unex.es (J.R.-M.); 2 Department of Medical-Surgical Therapy, Medicine and Health Sciences Faculty, Extremadura University, 06006 Badajoz, Spain; bertacaro@unex.es; 3 Mathematics Department, Medicine Faculty, Extremadura University, 06006 Badajoz, Spain; jmf@unex.es; 4 ROBOLAB Research Group, Medical-Surgical Therapy Department, Nursing and Occupational Therapy Faculty, Extremadura University, 10003 Cáceres, Spain; trdomin@unex.es

**Keywords:** cerebral palsy, children, international cooperation projects, orthopaedic disorders

## Abstract

Background: In international cooperation projects that are carried out in less developed and developing countries, a large number of children with disabilities present cerebral palsy (CP). Orthopaedic disorders are frequent complications associated with this disorder. Their prevention and early intervention are essential to achieve an appropriate therapeutic approach for children with PC and to improve their quality of life. Objective: To describe the treatment approach that is currently used in international cooperation projects for the rehabilitation management of the orthopaedic disorders in children with cerebral palsy. Methods: This is an observational, descriptive, cross-sectional study, carried out by means of an online questionnaire to professionals in the field of Physiotherapy and Rehabilitation working in international cooperation projects. The inclusion criteria were professionals working in the rehabilitation field in development aid, humanitarian action or emergency projects that provided rehabilitation services, working with children with cerebral palsy from 0 to 18 years old. Results: Ninety-eight questionnaires were analysed. The average age of the participants was 33.2 years, they were mainly working in development cooperation projects (83.33%) that were implemented in rehabilitation centres and through community-based rehabilitation services (60%). The projects were located in countries all over the world but mainly on the Asian continent (71.4%). Physiotherapists and orthopaedic technicians (72.22%) were the main professionals working in these projects, followed by occupational therapists and social workers (55.56%). The results indicated that the orthopaedic disorders were very frequent in the sample (66.67%), with hip subluxation (50%), scoliosis (77.78%), kyphosis (61.1%), clubfoot (88.7%) and varus foot (61.11%) standing out. The most commonly used treatment approaches were positioning (88.89%) and the Bobath concept (83.33%). The technical aids that were used by the professionals were ankle foot orthosis (AFO) (94.44%), bracing (66.67%), standing frames (83.33%), moulded seats (100%), corner seats (93.75%) and adapted seats (92.85%). Conclusions: In international cooperation projects, the rehabilitation treatment of children with cerebral palsy is based on a holistic approach. This is reflected in the interventions that are carried out to treat their orthopaedic disorders and in locally produced devices, awareness raising and community education. However, the professionals surveyed considered that the aids or orthoses used are insufficient in the treatment and prevention of orthopaedic disorders in cerebral palsy.

## 1. Introduction

Non-governmental organisations (NGOs) rely on experienced professionals to implement rehabilitation projects and achieve objectives in accordance with the requirements of international donors such as the European Commission’s Humanitarian Office [1,2]. This professionalisation leaves behind the image of the volunteer who goes to a developing country for short periods of time to carry out specific assistance work. Instead, it gives way to a professional who adapts his or her performance to the local context, focuses on the training of physiotherapists and rehabilitation professionals in the country and ensures the sustainability of the intervention [3,4,5].

The working system currently used in international cooperation projects in the field of disability is based on the twin-track approach. This approach addresses disability in a holistic way and employs strategies that focus on the personal needs of each person with a disability. In addition, it also considers the social and environmental factors that create disabling situations [6,7,8]. Rehabilitation projects implemented in the international cooperation field also follow the twin-track approach when planning and delivering their services. The working system varies in each country and each region according to the needs and particularities of the persons and the context [9,10]. This is why the active participation of persons with disabilities, their caregivers and their representative organisations are essential for the appropriate implementation of rehabilitation services [5,11].

The rehabilitation services for people with physical disabilities can be developed through three intervention units that can operate independently or in combination: Physical rehabilitation centre, where beneficiaries are registered and receive initial assessment and treatment, technical aids are distributed, information to beneficiaries and carers is given and referral to other health and social services in the community is done; mobile rehabilitation unit, which is formed by physiotherapists, rehabilitation assistants, occupational therapists, orthopaedic specialists, etc., and travels to communities and reference health centres to provide rehabilitation services; community based rehabilitation (CBR) which allows access to the services provided at the physical rehabilitation centre to those beneficiaries who live in remote areas and have difficulties to access the centre itself [5,12,13,14].

In cooperation projects carried out in less developed and developing countries, where the socio-economic reality is very disadvantaged, a large number of children with disabilities have cerebral palsy in all its forms [1,9,15]. Many of the beneficiaries of these projects access the rehabilitation services offered when the deformities and the orthopaedic disorders associated to the condition are already established. Therefore, the prevention and early treatment of these disorders is of vital importance as it will influence the quality of life of these children and their families [9,16].

The management of cerebral palsy requires continuous and appropriate rehabilitation that effectively meets the needs of both children and their parents or caregivers. In this respect, the literature indicates that the work being done in development aid projects may be the preferable method of service delivery in a context with limited resources [17,18]. However, the professionals involved in the rehabilitation services and in particular in the treatment of children with cerebral palsy, are confronted with difficult contexts that have limited health and social resources [5,19]. The treatment, technical aids and different orthopaedic devices and equipment used in these cooperation projects, as well as the follow-up of children with cerebral palsy must be adapted to many different aspects that need to be considered. These include the particular context where the project is implemented, the materials and local resources available (wood, foam, plaster, etc.) [9,15,16], the previous training of the rehabilitation professionals in the country, the geography of the place and the international non-governmental organisations involved [10,18].

David Werner in his book “Disabled children village” [20], presents in a simple but detailed way a treatment approach of cerebral palsy that is adapted to the context and needs of the poorest communities. This book is used as a working reference by physiotherapists and occupational therapists in cooperation projects all over the world as it provides, in a very illustrative way, resources and strategies for the treatment of cerebral palsy and its associated orthopaedic disorders.

Physiotherapy and rehabilitation projects are currently being implemented in development and humanitarian aid or emergency contexts in a large number of countries around the world [1,2,12,21,22,23]. These projects are providing services to adults and children with disabilities, achieving positive results and improving the quality of life of these people [1,21,22].

The assessments and the treatments carried out with children with cerebral palsy as well as the improvements or changes achieved are recorded and archived by the rehabilitation professionals [17,18]. However, there are no studies in the literature showing the results of the work being done in the non-governmental organisations. Therefore, the main objective of this study was to describe the therapeutic approach that is currently used in the international cooperation projects for the rehabilitation management of the orthopaedic disorders in cerebral palsy.

## 2. Materials and Methods

This is an observational, descriptive, cross-sectional study.

### 2.1. Ethical Aspects

All the ethical considerations and requirements mentioned in the Helsinki declaration [24] and the Data Protection Law [25] were met. The subjects included in the study signed the Informed Consent form to participate in the research.

### 2.2. Participants

The target population were professionals in the field of physiotherapy and rehabilitation who are currently working in international cooperation projects. The inclusion criteria established were professionals working in the rehabilitation field within development aid, humanitarian aid or emergency projects that provided rehabilitation service, to be working with children with cerebral palsy from 0 to 18 years old and to have given informed consent to participate in the study. The exclusion criteria were the absence of a signed informed consent, not working in the aforementioned rehabilitation field or not working with patients within the age range established.

### 2.3. Instrument

In order to obtain the research data, an online ad-hoc questionnaire was used in English and Spanish. It was developed by the authors of the study based on the most recent literature on the study subject. Databases such as Scielo, Medline, scientific search engines such as Pubmed and manual searches were consulted.

The self-administered questionnaire consisted of a total of 24 questions, divided into three sections. The first section collected sociodemographic and professional data. The second section was related to the therapeutic approach applied and included questions regarding the beneficiaries of the projects, the types of cerebral palsy, the orthopaedic disorders they presented and the type of treatment techniques, orthopaedic equipment and orthoses used in the projects. The third section focused on the bibliographic material used by the professionals in their daily practice.

### 2.4. Data Collection and Procedure

The data was obtained through the on-line questionnaire using the non-probabilistic snowball sampling method. The invitation to participate in the research was distributed by e-mail along with a link to an online questionnaire to different non-governmental organisations (NGOs). All participants were informed that their participation in the study was anonymous and voluntary and that no incentives would be offered. Together with the survey, information about the study, the use and protection of the data and the informed consent to participate in the research were enclosed. The participants could access the questionnaire online and the replies were downloaded and collected in an excel sheet. Data were stored in an encrypted computer and only the authors had access to the information during all stages of the study. The questionnaire was available from March to May 2019 and once this period of time was over, the data collection was completed.

### 2.5. Data Analysis

The analysis of the data collected in the completed questionnaires was carried out with the statistical programme IBM SPSS Statistics for Windows, Version 22.0. (Armonk, NY, USA: IBM Corp). All responses were coded to ensure the anonymity of the participants and were analysed by staff that were external and independent to the study. Due to the type of outcome measures, which were mostly qualitative, a descriptive study of the variables analysed was carried out with the percentages and frequency distributions. In addition, the relationships between outcomes were analysed through the χ^2^ ant *t*-test, considering 5% as significant level.

## 3. Results

A total of 103 responses were received out of which 98 were valid. Five questionnaires were excluded because they did not meet the inclusion criteria established in the study.

### 3.1. Socio-Demographic and Professional Data

The mean age of the participants was 33.19 ± 2.68. From the total sample, 77.8% of the participants were men while 22.2% were women. We did not find out significant differences in mean between women’s and men’s age. The majority of the participants (69.4%) worked as physiotherapists, 25.5% as occupational therapists and 5.1% as prosthetics and orthotics technicians. Most men (76.3% of them) worked as physiotherapists, while women distributed equally between physiotherapists (45.5%) and occupational therapists (50%). The statistical analysis showed that this association between gender and profession was significant (*p* = 0.011).

The projects in which the participants worked were mainly in a development aid context (83.3%) and to a lesser extent in emergency and humanitarian aid (16.7%). In general, the rehabilitation services of the projects were provided in hospitals (11.1%), in rehabilitation centres (22.2%), through community-based rehabilitation (16.7%) and in these two in parallel (50% of the total).

Out of the total number of development projects, 13.33% were carried out in rehabilitation centres, 20% through community-based rehabilitation services, 60% in both simultaneously and 6.7% in hospitals. Regarding the emergency and humanitarian aid projects, 66.6% of them were implemented in rehabilitation centres and 33.3% in hospitals.

The teams of professionals working on the projects were mostly multidisciplinary (94.4%). The other health professionals that were part of the team were physiotherapists and prosthetics and orthotics technicians (72.2% each), occupational therapists (55.6%), social workers (55.6%), rehabilitation assistants (44.4%), speech therapists (22.2%), doctors and teachers (16.7% each), psychologists (5.6%) and other professionals such as community workers (16.7%).

The countries in which the projects were being implemented are distributed all over the world. Of all these countries, 71.4% belong to the Asian continent, 17.3% to the African continent and 11.3% to America. Of the total number of physiotherapists, the majority worked in Asia (67.6%), all occupational therapists worked in Asia and all prosthetics and orthotics technicians worked in Africa. This association was significant (*p* < 0.001). Table 1 shows all the data of the first section of the questionnaire.

### 3.2. Beneficiaries Receiving Rehabilitation Services

Table 2 provides the data related to the beneficiaries of the projects, the types of cerebral palsy, the orthopaedic disorders that they had. Since treating children with cerebral palsy was an inclusion criteria of the study, all the professionals that completed the questionnaire were treating children with cerebral palsy in their rehabilitation services. All the projects treated children under 12 and 73.5% of them also treated children between 13 and 18 years old.

A total of 83.33% of the total number of professionals who participated in the study responded that more than 20 children with cerebral palsy were receiving treatment in their projects. A lower percentage of professionals were providing services to between 11 and 20 children (11.11%) and between 1 and 10 children (5.56%).

With regard to the type of cerebral palsy and according to their motor impairment, we can highlight that spastic cerebral palsy was the most treated by the professionals (94.44%), followed by athetoid cerebral palsy (77.78%). On the other hand, according to the anatomical distribution of the lesion, 77.8% presented hemiplegia, 50% presented diplegia and 44.44% presented tetraplegia.

### 3.3. Orthopaedic Disorders Associated to Cerebral Palsy

The data showed that all respondents reported that children with cerebral palsy who are treated in their projects have orthopaedic disorders associated with their condition. Regarding the orthopaedic disorders of the hip, the most frequent was the subluxation (50%). Concerning the orthopaedic disorders of the spine, 77.78% of the children treated in the projects had scoliosis. Other spinal disorders such as kyphosis or lumbar hyperlordosis were present in 61.11% and 50% of the children, respectively. The most frequently identified foot disorder was clubfoot (88.89%), followed by varus foot (61.11%).

It should be noted that 44% of the participants considered that the children they treat have other types of disorders not included in the questions of the questionnaire such as contractures and deformities at the hip, knee, ankle, elbow and toes; joint stiffness of the knee and ankle; shortening of the muscles and specially the hip adductor muscles; knee flexion, genu recurvatum and genu valgum and coxa vara and valga.

### 3.4. Treatment Techniques

The data related to the treatment techniques used for the management of cerebral palsy in the international cooperation projects is shown in Table 3. The most commonly used treatment technique was positioning, being applied in 88.9% of the projects. Motor development facilitation and the Bobath concept were the next most used techniques (83.3% both). These techniques were followed by active mobilisations and stretching (77.78%), passive mobilisations (72.2%), resisted mobilisations (22.2%) and the Le Métayer and Vojta concept approaches (11.1%). A total of 22.2% of the participants responded that they use other treatment techniques. These include functional exercises and activities of daily living training, exercises to work on cognitive and perceptual skills, fine and gross motor activities, proprioceptive neuromuscular facilitation, coordination and balance exercises.

With regard to the technical aids, assistive devices and orthotic devices used in the different projects for the treatment and prevention of orthopaedic disorders, the most commonly used foot orthosis is the ankle foot orthosis (AFO) (94.8%), followed by the dynamic ankle foot orthosis (DAFO) (38.9%). The spinal braces were part of the therapeutic approach in 66.7% of the projects. Regarding the seating devices, corner seats and adapted seats were the most common, being used in 88.7% and 77.8% of the projects respectively. In contrast, moulded seats were only used in 33.3% of projects. A large number of projects used standing frames in their therapeutic approach, corresponding to the 83.3% of the total number of projects. A total of 55.6% of the participants responded that they use other technical aids or orthoses, which include knee ankle foot orthosis (KAFO), hip, knee, ankle and foot orthoses, upper limb orthoses, orthoses and technical aids for the activities of daily living, night orthoses for knee extension, orthopaedic shoes and mobility technical aids.

Among the types of adapted seating devices, we could observe that the most used was the toilet seat (61.11%). A total of 11.1% of the participants reported that they use home chairs in addition to the toilet seat. No participants had children with cerebral palsy using school chairs and 27.8% did not give an answer regarding the adaptive seats.

The technical aids, assistive and orthotic devices used in the projects were mainly manufactured locally, i.e., in the locality where the cooperation project was implemented. The results varied depending on the type of orthopaedic equipment. In the case of the special seats, the three types were mostly locally produced. The moulded seats were locally made in 100% of the projects, the corner seats in 93.7% and the adapted seating devices in 92.8%. In relation to the spinal braces, they were manufactured locally in 50% of the projects that included them in their therapeutic approach. The AFOs and DAFOs were produced locally in 64.7% and 57.1% of the projects that prescribed them for their beneficiaries with cerebral palsy, respectively. Regarding the standing frames, in 92.8% of the projects that use them, they were locally made.

The results showed that 83.3% of the projects had an orthopaedic workshop or worked in collaboration with a prosthetics and orthotics technician or with a workshop in the local area. In contrast, 16.7% did not have this facility.

All projects included in the study provided information and training on positioning and prevention of orthopaedic disorders to the family members or the caregivers of the children with cerebral palsy, as well as to the children themselves when possible. The type of information was mostly through hands-on training sessions (88.9%) and informative talks (88.9%). It should be highlighted that no cooperation project used audio-visual media (videos) for the caregiver’s training. Physiotherapists were mainly the professionals who provide the families or caregiver’s training and information (77.8%), followed by the occupational therapists (57.1%). In comparison doctors registered a low percentage regarding their implication on this training and awareness raising activity (5.6%).

All the professionals surveyed indicated that they have to take into account the local context when implementing their projects and providing treatment to children with cerebral palsy. They highlighted the importance of the socio-economic factors (83.7%), the budget (53.0%), the cultural aspects (53.0%) and other aspects (76.5%). These other aspects included the local geography (distances, nomadic regions, etc.), the education and qualifications of the local staff, health, the parents’ expectations of rapid healing or improvement and the need to use locally available materials to ensure sustainability of technical aids and services to beneficiaries and their caregivers.

It should also be noted that 56.1% of the respondents considered that the therapeutic approach and the technical aids or orthoses used in their rehabilitation services were insufficient for the treatment and prevention of orthopaedic disorders in children with cerebral palsy. This outcome correlated with age, since the critical group was 1.1 older in average (*p* = 0.045). It did not correlate with profession and country. However, it showed a significant relationship with gender (*p* = 0.034). In fact, 63.6% of men in study considered the methods to be sufficient while only 38.2% of women did.

### 3.5. Reference and Bibliographic Resources

**Figure 1:**
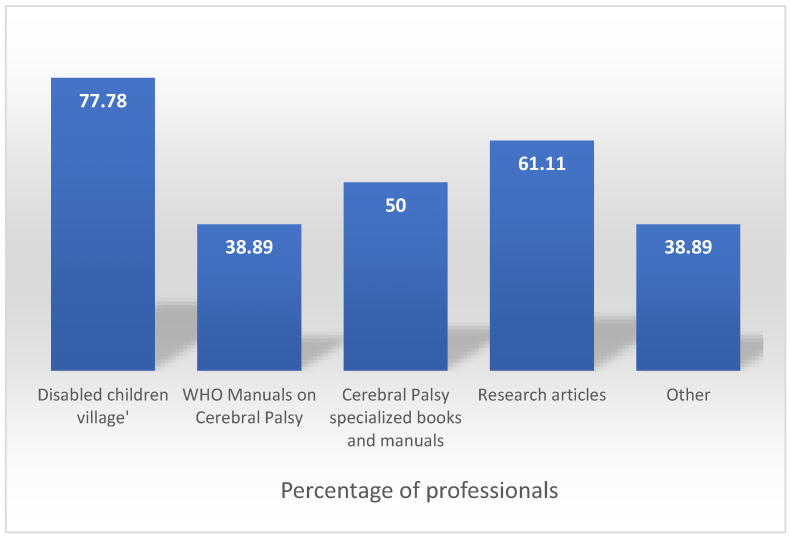
Percentage of professionals using different bibliographic resources.

All the professionals reported that they based their therapeutic approach to cerebral palsy and the associated orthopaedic disorders on bibliographic resources. Figure 1 shows the percentage of professionals that used the different resources included in the questionnaire. David Werner’s book, ‘Disabled Children Village’ is the most used (77.8%), followed by research articles (61.1%), manuals and books specialising in cerebral palsy (50%).

## 4. Discussion

The main objective of this study was to describe the rehabilitation treatment approach that is currently used in international cooperation projects for the management of the orthopaedic disorders in children with cerebral palsy.

The importance of prevention and early treatment of the orthopaedic disorders associated with cerebral palsy is well known [26,27]. There is current evidence that supports the effectiveness of different rehabilitation treatments available for the management of these disorders. In this regard, Poutney et al. [27,28] demonstrated that the use of a postural management programme using the Chailey Adjustable Positioning System (CAPS) which included a lying support system, an adapted seat and a standing frame had a significant influence on hip disorders. It decreased the incidence of hip disorders and the need for surgical intervention and botulinum toxin treatment. Picciolini et al. [29] confirmed the benefits of the application of a plaster moulded seat in combination with physiotherapy (Bobath concept approach). Their results showed a progressive reduction of the hip displacement in the experimental group. In addition, Jain et al. [30] also highlighted the advantages of the use of materials such as plaster. This material has a low cost and is easy and safe to apply which allows postural management devices to be made even in remote places with minimal facilities available. In addition, the findings of the study conducted by MacDonald et al. [31] evidenced that a seating system with knee blocks and a sacral pad may improve hip position in children with cerebral palsy.

We coincide with the different studies found in the literature in use the adapted and moulded seats for the management and prevention of hip disorders in children with cerebral palsy [27,28,29]. However, we cannot contrast the use of corner seats with any existing results as no studies on this topic have been found in the literature. The high percentage of adapted toilet seats that are manufactured in the rehabilitation projects can be explained by the presence of latrines in the toilets of disadvantaged communities. Children with cerebral palsy face difficulties in maintaining their toilet position and require special seating to provide the necessary support.

Standing frames are used in a high percentage of the projects, which is consistent with the findings of other research such as that of Poutney et al. [27,28]. The use of these devices in the management of cerebral palsy is also supported by the Le Métayer concept. Le Métayer considers that weight bearing is essential for the prevention of hip disorders and to improve hip joint integrity [32].

In relation to the rehabilitation treatment of spinal disorders, we can highlight that Vekerdy [33] evidenced that the use of a special seating device (a thoracic-lumbar-sacral orthosis with non-rigid SIDO frame) reduced kyphosis and improved posture. These improvements also had a positive effect on feeding and upper limb coordination of the children with cerebral palsy. Regarding the application of braces, Touzeau [34] affirmed that the Garchoi brace is currently the most suitable trunk support for the child with cerebral palsy. Our results are consistent with the aforementioned studies.

The scientific research has demonstrated the efficacy of the assistive and orthotic devices for the prevention and the conservative treatment of orthopaedic disorders in infantile cerebral palsy. Diverse studies have shown improvements in the range of movement, the gait efficiency and the gait pattern after the use of the AFOs by children with cerebral palsy having associated foot disorders [35,36,37,38]. Buckon et al. [36] also found a decrease in energy expenditure during gait with the use of AFOs. In contrast, Rogozinski et al. [37] noted that the presence of knee and hip contractures greater than 15° are contraindications to the use of the ground reaction AFO. In addition, Bennett et al. [38] concluded that AFOs decrease gait effort but not in all children with cerebral palsy. Lam et al. [39] compared the effect of the AFO and the DAFO and found that both orthosis have positive effects on the gait of children with this condition. After analysing the results of our research, we consider that they are consistent with the published research that study the use of AFOs and DAFOs. The AFO is an orthosis device that is frequently used in cooperation projects and is mainly manufactured in the local area where the projects are being implemented. The DAFOs is, however, used to a lesser extent. This may be due to the greater complexity of manufacturing these orthoses and the need for a greater variety of materials, which makes them more expensive.

Therefore, the treatment approach applied for the rehabilitation of orthopaedic disorders associated to cerebral palsy in international cooperation projects coincides with the treatment approach described in the literature. Despite the fact that the data obtained showed that only 11.11% of the participants used the orthopaedic equipment proposed by the Le Métayer school [40,41], we consider that they could be used to a greater extent. This statement is based on the fact that the Le Métayer assistive devices are made of materials that are easily available, have a low cost, can be easily transported and can be easily remodelled or modified in case of need due to growth or changes in the condition of children with cerebral palsy. We consider that the increase of the use of these devices could improve the rehabilitation and prevention of the orthopaedic disorders in children with cerebral palsy that receive rehabilitation services in development and emergency aid projects.

According to the report on disability of the World Health Organisation (WHO) [42], technical aids should be adapted to the user and their environment and appropriate follow-up should be carried out to ensure the safe and efficient use of the different orthopaedic equipment. This is reflected in our results as the participants that completed the questionnaire reported the importance and the need to consider particular aspects of the context when providing rehabilitation services.

Although the teams of professionals working on the projects were mostly multidisciplinary (94.4%), there were higher percentages of physiotherapists and prosthetics and orthotics technicians (72.2% each), occupational therapists (55.6%), social workers (55.6%) and rehabilitation assistants (44.4%) in comparison to other professionals such as speech therapists (22.2%), doctors and teachers (16.7% each), psychologists (5.6%) and community workers (16.7%). A multidisciplinary approach is essential for the appropriate management of children with cerebral palsy [43,44]. Different professionals like paediatricians, neuro, general and orthopaedic surgeons, physiotherapists, occupational therapists, speech and language therapists, psychotherapists, orthotists and nursing specialists, teachers and social workers should work together to achieve a positive effect of the treatments applied, reducing physical impairment of the children [43,44]. Therefore, we can highlight that our results show that the presence of the medical staff is insufficient. In particular, the orthopaedic surgeon has a key role as children with cerebral palsy often develop secondary progressive orthopaedic conditions and deformities that may require surgical treatment [45,46]. In our study, we have focused on the rehabilitation treatment and therefore, organizations specialized in the rehabilitation part of the management of cerebral palsy were contacted. The projects were implemented mainly in rehabilitation centres and community-based rehabilitation settings. Only 11.1% of the projects were implemented in hospitals and this maybe the reason why there was a low percentage of doctors in the multidisciplinary teams. Orthopaedic surgeons may have been involved in the treatment of ortopaedic disorder in the children with cerebral palsy of the projects but, based on our experience as expatriates in different developing and emergency rehabilitation projects, we sense that the patients encounter many difficulties to have access to surgery and to receive the appropriate post-operative care [45].

The difficulties that children with cerebral palsy and their families have to face and which prevents them from receiving comprehensive treatment are directly related to economic difficulties. In less developed and developing countries, there is a lack of local funding for health care due to the disadvantaged socio-economic reality [1,5,19]. In consequence health professionals involved in the treatment of children with cerebral palsy, are confronted with difficult contexts that have limited health and social resources [5,19]. In fact, shortages of health personnel are still very large in some countries. In Africa, for example, the number of doctors is 2.5 per 10,000 inhabitants and the number of nurses is 9.1 per 10,000. In addition, in terms of infrastructure, the continent has an average of less than one hospital per 100,000 inhabitants [47]. Therefore, the treatment that children with cerebral palsy receive in less developed and developing countries are often not complete. In this respect, surgical interventions that are essential to optimise physical function and help the prevention of deformities [46,48], may be compromised due to the difficulties of the patients and their families to have access to these services. In addition, if surgical interventions are performed without the appropriate multidisciplinary medical and rehabilitation team and within health facilities that do not meet all the requirements for a proper intervention and follow up, the outcomes of the interventions could cause the worsening of the orthopaedic disorders of cerebral palsy [46].

The global economic crisis has also affected the functioning of NGOs regardless of their size or characteristics. The financial resources allocated to the health sector do not seem to meet the needs of the countries in need of help, which is particularly detrimental in this sector [47]. The dismantling of social welfare or aid budgets in the most affected countries has compromised their ability to raise funds to develop projects both locally and in other countries [47]. In this respect, the key recommendations of the WHO report include, among other measures, better development of training curricula for health professionals and an international commitment to support countries most affected by the lack of health professionals [49]. Health is the key element of all health systems and is fundamental to the improvement and progress of the health of the population [50,51].

As already mentioned, appropriate management of children with cerebral palsy would be possible only with a multi/interdisciplinary team approach. However, the child and the family should be the centre of all the decision-making process [43]. Families are the world expert on their child [43] and they are a key component for the improvement of their child. Despite this, parents’ expectations of their children’s development are not well known, as published information on parental expectations is scarce. The scientific literature available indicates that the parents of children with this condition have low expectations in relation to their future independence and success [52,53]. In relation to the services that can improve the lives of families with children with cerebral palsy, the families feel dissatisfaction and frustration [54]. These data are in line with our results as 56.1% of the respondents considered that the therapeutic approach and technical aids or orthoses used in their rehabilitation services were insufficient for the treatment and prevention of orthopaedic disorders in children with cerebral palsy.

In order to minimize the frustration of parents and to help them to adjust their expectations to the reality, awareness raising is a key aspect. Our results indicate that rehabilitation staff provide information on orthopaedic disorders associated to cerebral palsy and their treatment to family members or caregivers. This training was performed mainly by physiotherapists and occupational therapists and was mainly conducted through hands-on training and information talks (88.9%). In addition, this training was supported by leaflets in the 61.1%. Our study shows results in relation to the orthopaedic disorders associated to cerebral palsy, but it would be interesting to deepen into this aspect and find out more about the training conducted to raise awareness on cerebral palsy, its clinical features and the progression of the condition.

### Limitations of the Study

The fact that the survey was done through an online questionnaire sampling method allowed us to reach a large sample. However, it also made it more difficult for the researchers to control the sample and important information for the research may have been lost. We consider that some questions about the use of any additional devices, the medical and surgical team involved in the treatments or the surgery interventions received by the children could have been included in the questionnaire making it more complete. Nevertheless, they were not included as this would have implied a much larger questionnaire increasing the risk of the participants not completing the surveys due to the length of the questionnaire.

Another possible limitation could have been the possible misunderstandings when completing the survey as the mother tongue of some participants was not English or Spanish, the languages in which our tool was designed. We believe that future research should take these considerations into account.

This study has focused on the rehabilitation treatment of the orthopaedic disorder associated to cerebral palsy and therefore some aspects of the comprehensive multidisciplinary treatment that the children with cerebral palsy need may be missed. In particular, the medical and surgical treatment is a key aspect of the management of these disorders, but no information related to these specialties have been included in this study. Therefore, we consider that adding the information related to these specialities would be essential to understand the global scenario in developing countries regarding the comprehensive treatment of children with cerebral palsy.

## 5. Conclusions

In the international cooperation projects surveyed, the rehabilitation services provide a comprehensive rehabilitation treatment of the orthopaedic disorders of children with cerebral palsy that is consistent with the scientific evidence. This is reflected in the management of these disorders which includes the use of technical aids, assistive and orthotic devices that are locally made, the use of rehabilitation treatment techniques, caregiver’s training, awareness raising and community education. However, most of the professionals working in these cooperation projects consider that the therapeutic approach and the technical aids or orthosis used are insufficient in the treatment and prevention of orthopaedic disorders in cerebral palsy.

## Figures and Tables

**Table 1:** Socio-demographic and professional data.

Outcomes	Options	*N* (%)
Gender	Female Male	22 (22.2) 76 (77.8)
Profession	Physiotherapist Occupational Therapist Prosthetics and Orthotics Technician	68 (69.4) 25 (25.5) 5 (5.1)
Current position in the rehabilitation project	Physiotherapist Occupational Therapist Trainer/teacher Assessor Project manager/Project coordinador Rehabilitation service manager/coordinator Rehabilitation facilitator Head of department	27 (27.8) 5 (5.6) 11 (11.1) 11 (11.1) 22 (22.2) 5 (5.6) 11 (11.1) 5 (5.6)
Type of project	Development aid Emergency and humanitarian aid	82 (83.3) 16 (16.7)
Place where the rehabilitation services are provided	Hospital Rehabilitation centre Community based rehabilitation Rehabilitation centre and Community based rehabilitation	11 (11.1) 22 (22.2) 16 (16.7) 49 (50.0)
Professionals included in the multidisciplinary team	Physiotherapist Occupational therapist Prosthetics and Orthotics Technician Social worker Rehabilitation assistant Speech therapist Psychologist Doctor Teacher Community worker	71 (72.2) 76 (77.2) 55 (55.6) 55 (55.6) 44 (44.4) 22 (22.2) 5 (5.6) 16 (16.7) 16 (16.7) 16 (16.7)
Country where the projects are implemented	Africa Asia America	17 (17.3) 70 (71.4) 11 (11.3)
Professionals by continent where the projects are implemented	Physiotherapist	Africa 16 (16.9)
		Asia 66 (67.6)
		America 15 (15.5)
	Occupational Therapist	Africa 0
		Asia 98 (100)
		America 0
	Prosthetics and Orthotics Technician	Africa 98 (100)
		Asia 0
		America 0

**Table 2:** Characteristics of the patients, type of cerebral palsy and types of associated orthopaedic disorders.

Outcomes	Options	*N* (%)
Age	0–12 years 13–18 years	98 (100) 72 (73.5)
Number of children that receive rehabilitation services in the project	1–10 children 11–20 children More than 20 children	5 (5.6) 11 (11.1) 82 (83.3)
Type of cerebral palsy according to the motor disorder	Spastic Ataxic Athetoid Mixed	93 (94.4) 54 (55.6) 76 (77.8) 65 (66.7)
Type of cerebral palsy according to the anatomical affected area	Hemiplegic Diplegic Tetraplegic	76 (77.8) 49 (50.0) 44 (44.8)
Frequency of orthopaedic disorders in the children with cerebral palsy	Never Not very often Very often	0 33 (33.3) 65 (66.7)
Types of orthopaedic hip disorders in children with cerebral palsy	Hip subluxation Hip luxation	49 (50.0) 33 (33.3)
Types of orthopaedic spine disorders in children with cerebral palsy	Scoliosis Kyphosis Lumbar hiperlordosis	76 (77.8) 60 (61.1) 49 (50.0)
Types of orthopaedic foot disorders in children with cerebral palsy	Equinus Talus Varus Valgum Flat feet	87 (88.9) 33 (33.3) 60 (61.1) 49 (50.0) 54 (55.6)

**Table 3:** Treatment approaches used for the management of cerebral palsy and its associated orthopaedic disorders in international cooperation projects.

Outcomes	Options	*N* (%)
Rehabilitation treatment approaches used	Passive mobilisations Active mobilisations Resisted mobilisations Stretching Positioning Motor development facilitation or neurodevelopmental treatment techniques Bobath Concept Le Métayer Concept Vojta Concept Other	71 (72.2) 76 (77.8) 22 (22.2) 76 (77.8) 87 (88.7) 82 (83.3) 82 (83.3) 11 (11.1) 11 (11.1) 22 (22.2)
Technical aids, assistive and orthotic devices used	Moulded seats Corner seats Adapted seats Spinal brace AFO DAFO Standing frame Other	33 (33.3) 87 (88.9) 76 (77.8) 65 (66.7) 93 (94.8) 38 (38.9) 82 (83.3) 54 (55.1)
Adapted seats used	Toilet seat School chair Toilet seat and home chair No answer	60 (61.1) 0 11 (11.1) 27 (27.8)
Locally made technical aids, assistive devices and orthosis	Moulded seat Corner seat Adapted seat Spinal brace AFO DAFO Standing frame	98 (100) 93 (93.7) 92 (92.8) 49 (50.0) 63 (64.7) 56 (57.1) 91 (92.8)
Information on positioning and orthopaedic disorders provided to family members and beneficiaries	Hands-on training Information talk Leaflets Videos Other (posters)	87 (88.9) 87 (88.9) 60 (61.1) 0 5 (5.6)
Professionals in the team providing information on orthopaedic disorders and their treatment to family members/caregivers	Occupational therapist Physiotherapist Prosthetics and Orthotics Technician Rehabilitation assistant Social worker Doctor Speech therapist	56 (57.1) 76 (77.8) 44 (44.9) 49 (50.0) 27 (27.5) 5 (5.6) 11 (11.1)
Aspect of the local context considered when treating children with cerebral palsy	Socio-economic Budget Cultural Other	82 (83.7) 52 (53.0) 52 (53.0) 75 (76.5)
Do you consider that the treatment approach and devices that you use are enough and efficient on the treatment and prevention of orthopedic complications in Cerebral Palsy?	Yes No	43 (43.9) 55 (56.1)

Note: AFO: Ankle foot orthosis; DAFO: Dynamic ankle foot orthosis.

## Data Availability

The data underlying this article cannot be shared publicly to maintain the privacy of individuals that participated in the study. The data will be shared on reasonable request to the corresponding author.

## References

[1] Llorente P. (2017). What situation does physiotheraphy face in Mozambique? The FISIAFRICA Project. Gac. Sanit..

[2] Stucki G., Zampolini M., Juoce A. (2017). Practice, science and governance in interaction: European effort for the system-wide implementation of the International Classification of Functioning, Disability and Health (ICF) in physical and rehabilitation medicine. Eur. J. Phys. Rehabil. Med..

[3] Lysack C., Krefting L. (1993). Community-based rehabilitation cadres: Their motivation for volunteerism. Int. J. Rehabil. Res..

[4] Gellert G., Walsh W., Petrosia L. (1995). Role of voluntarism and nongovernmental organizations in internationalizing rehabilitation medicine. Am. J. Phys. Med. Rehabil..

[5] Ying L., Hongmei M. (2021). Interorganisational cooperation and its effects on community rehabilitation for people with severe mental disorders in Beijing, China: A case study. Health Soc. Care Community.

[6] Schranz B., Shirivastaba A., Mohapatra V., Ranjan A. (2009). Mainstreaming Disability in Community based Disaster Risk Reduction.

[7] Corry P. (2004). A twin track approach. Nurs. Stand..

[8] Manning B., Benton S. (2008). The safe implementation of research into healthcare practice. Stud. Health Technol. Inf..

[9] Namaganda L., Kobusingye O., Olikira S., Mayora C., Bentley J. (2017). Disability characteristics of community-based rehabilitation participants in Kayunga district, Uganda. Ann. Glob. Health.

[10] Fonseca L., Almeida C. (2015). International cooperation and health policy implementation in a post-conflict situation: The case of East Timor. Hist. Cienc. Saude. Manguinhos.

[11] Raja S., Boyce W., Ramani S., Underhill C. (2008). Success indicators for integrating mental health interventions with community-based rehabilitation projects. Int. J. Rehabil. Res..

[12] World Health Organization (2003). International Consultation to Review Community-Based Rehabilitation (CBR).

[13] Robertson J., Emerson E., Hatton C., Yasamy M. (2012). Efficacy of community-based rehabilitation for children with or at significant risk of intellectual disabilities in low- and middle-income countries: A review. J. Appl. Res. Intellect. Disabil..

[14] Crishna B. (1999). What is community-based rehabilitation? A view from experience. Child Care Health Dev..

[15] Groote W. (2019). Concept changes and standardizing tools in community-based rehabilitation. Phys. Med. Rehabil. Clin. N. Am..

[16] Penny N., Zulianello R., Dreise M., Steenbeek M. (2007). Community-based rehabilitation and orthopaedic surgery for children with motor impairment in an African context. Disabil. Rehabil..

[17] Dambi J., Jelsma J. (2014). The impact of hospital-based and community based models of cerebral palsy rehabilitation: A quasi-experimental study. BMC Pediatr..

[18] Hurley D., Sukal T., Msall M., Gaebler D., Drosschell K., Devald J. (2011). The cerebral palsy research registry: Development and progress toward national collaboration in the United States. J. Child Neurol..

[19] Turmusani M., Vreede A., Wirz S.L. (2002). Some ethical issues in community-based rehabilitation initiatives in developing countries. Disabil. Rehabil..

[20] Werner D. (1999). Disabled Children Village.

[21] Steihaug S., Lippestad J., Isaksen H., Werner A. (2014). Development of a model for organisation of and cooperation on home-based rehabilitation—An action research project. Disabil. Rehabil..

[22] Konno K., Chibwana S., Takata Y. (2019). Intensive physiotherapy with subsequent community-based rehabilitation: Two cases of cerebral malaria in rural areas of Malawi. J. Phys. Sci..

[23] Patterson A., Bahle-Lampe A., Greiner B., Bracciano A., Lohman H., Mu K., Qi Y. (2020). Meeting global rehabilitation needs: The development and evaluation of an international visiting rehabilitation student program. J. Allied Health Summer.

[24] Asociación Médica Mundial (2001). Declaración de Helsinki de la Asociación Médica Mundial. Principios éticos para las investigaciones médicas en seres humanos. Anales del Sistema Sanitario de Navarra.

[25] (2016). Regulation (EU) 2016/679 of the European Parliament and of the Council of 27 April 2016 on the protection of individuals with regard to the processing of personal data and on the free movement of such data and repealing Directive 95/46/EC (General Data Protection Regulation). Off. J. Eur. Union.

[26] García E., Capablo B. (1999). Valoración y estudio de las deformidades ortopédicas en personas con parálisis cerebral. Fisioterapia.

[27] Pountney T., Mandy A., Green E., Gard P. (2002). Management of hip dislocation with postural management. Child Care Health Dev..

[28] Pountney T.E., Mandy A., Green E., Gard P.R. (2009). Hip subluxation and dislocation in cerebral palsy—a prospective study on the effectiveness of postural management programmes. Physiother Res. Int..

[29] Picciolini O., Gasparroni V., Cozzaglio M., Messina L., Portinaro N., Mosca F. (2010). Le centrage des hanches au moyen de sièges moulés—études et résultats. Mot. Cereb. Readapt. Neurol. Dev..

[30] Jain S., Mathur N., Joshi M., Jindal R., Goenka S. (2008). Effect of serial casting in spastic cerebral palsy. Indian J. Pediatr..

[31] McDonald R.L., Surtees R. (2007). Longitudinal study evaluating a seating system using a sacral pad and kneeblock for children with cerebral palsy. Disabil. Rehabil..

[32] Esparza T. (2006). Férula Pelvipédica. Libro de Ponencias de las XVI Jornadas de Fisioterapia: Fisioterapia y Parálisis Cerebral.

[33] Vekerdy Z. (2007). Management of seating posture of children with cerebral palsy by using thoracic-lumbar-sacral orthosis with non-rigid SIDO frame. Disabil. Rehabil..

[34] Touzeau C. (2007). Traitement orthopédique par corset garchois: Quand, comment, pourquoi?. Mot. Cereb. Readapt. Neurol. Dev..

[35] Brehm M.A., Harlaar J., Schwartz M. (2008). Effect of ankle-foot orthoses on walking efficiency and gait in children with cerebral palsy. J. Rehabil. Med..

[36] Buckon C.E., Thomas S.S., Jakobson S., Moor M., Sussman M., Aiona M. (2004). Comparison of three ankle-foot orthosis configurations for children with spastic diplegia. Dev. Med. Child Neurol..

[37] Rogozinski B.M., Davids J.R., Davis R.B., Jameson G.G., Blackhurst D.W. (2009). The efficacy of the floor-reaction ankle-foot orthosis in children with cerebral palsy. J. Bone Jt. Surg. Am..

[38] Bennett B.C., Russell S.D., Abel M.F. (2012). The effects of ankle foot orthoses on energy recovery and work during gait in children with cerebral palsy. Clin. Biomech..

[39] Lam W.K., Leong J.C., Li Y.H., Hu Y., Lu W.W. (2005). Biomechanical and electromyographic evaluation of ankle foot orthosis and dynamic ankle footorthosis in spastic cerebral palsy. Gait Posture.

[40] Le Metayer M. (1995). Reeducación Cerebromotriz del Niño Pequeño.

[41] Le Métayer M. (2004). Le “Trotte-lapin” amélioré. Mot. Cerebrale Readapt. Neurol. Dev..

[42] World Health Organization (2011). World Report on Disability.

[43] Fairhurst C. (2012). Cerebral palsy: The whys and hows. Arch. Dis. Child. Educ. Pract. Ed..

[44] Gulati S., Sondhi V. (2018). Cerebral Palsy: An Overview. Indian J. Pediatr..

[45] Patel D.R., Neelakantan M., Pandher K., Merrick J. (2020). Cerebral palsy in children: A clinical overview. Transl. Pediatr..

[46] Sharan D. (2017). Orthopedic surgery in cerebral palsy: Instructional course lecture. Indian J. Orthop..

[47] García Raya M.E., Tapia Domínguez E. (2015). La salud en el post 2015 ¿Mucho ruido y pocas nueces?. Salud y Cooperación Para el Desarrollo: Análisis Constructivo y Nuevas Claves de Futuro.

[48] Novak I., Morgan C., Fahey M., Finch-Edmondson M., Galea C., Hines A., Langdon K., Mc Namara M., Cb Paton M., Popat H. (2020). State of the Evidence Traffic Lights 2019: Systematic Review of Interventions for Preventing and Treating Children with Cerebral Palsy. Curr. Neurol. Neurosci. Rep..

[49] OMS: Informe Sobre la Salud En el Mundo 2006—Colaboremos Por la Salud. www.who.int/whr/2006/es/index.html.

[50] Casamitjana N., Alonso P.L. (2007). El papel de la investigación y la formación en la cooperación en salud. JANO Extra Oct..

[51] Torrús Tendero D. (2011). Investigación y Cooperación en Salud Internacional. Enferm. Emerg..

[52] Barak S., Elad D., Silberg T., Brezner A. (2017). Mothers and fathers of children with cerebral palsy: Differences in future expectations. J. Dev. Phys. Disabil..

[53] Magill-Evans J., Darrah J., Pain K., Adkins R., Kratochvil M. (2001). Are families with adolescents and young adults with cerebral palsy the same as other families?. Dev. Med. Child Neurol..

[54] Darrah J., Magil-Evans J., Adkins R. (2002). How well are we doing? Families of adolescents or young adults with cerebral palsy share their perceptions of service delivery. Disabil. Rehabil..

